# Minnesota Hospital College

**Published:** 1888-07

**Authors:** 


### Minnesota Hospital College—Dental Department.

The seventh annual commencement of the Dental Department of the Minnesota Hospital College was held, in conjunction with that of the Medical Department, in the Hennepin-avenue M. E. Church, Minneapolis, Minn., on Friday, March 16, 1888.

The degree of D.D.S. was conferred on the following graduates by C. H. Hunter, A.M., M.D., President of the Faculty:

| NAME. | RESIDENCE. | NAME. | RESIDENCE. |
|---|---|---|---|
| H. G. Dampier | Minnesota. | C. L. Sargent | Wisconsin. |
| A. N. Cheney | Minnesota. | D. H. Carpenter | Minnesota. |
| C. D. Snow | Minnesota. | A. H. Benson, M.D. | Wisconsin. |
| H. T. Burnette | Minnesota. | J. D. Jewett | Minnesota. |

